# An Uncommon Diagnosis in a Common Presentation: Renal Vein Thrombosis in a Young Adult

**DOI:** 10.7759/cureus.94651

**Published:** 2025-10-15

**Authors:** Brandon J Hegele, Philip E Sisser, Parvathi Perumareddi

**Affiliations:** 1 College of Medicine, Florida Atlantic University Charles E. Schmidt College of Medicine, Boca Raton, USA; 2 Family Medicine, Florida Atlantic University Charles E. Schmidt College of Medicine, Boca Raton, USA

**Keywords:** complicated urinary tract infection, nephrotic syndrome, nicotine use, oral contraceptives, renal vein thrombosis, venous thromboembolism (vte)

## Abstract

Renal vein thrombosis (RVT) is a condition primarily associated with nephrotic syndrome and malignancy, with a mean age of occurrence at 55 years. There are a few documented cases of RVT in the setting of a complicated urinary tract infection (UTI), especially in younger patients. We aim to examine the case of a 21-year-old female who presents with RVT in the setting of multiple risk factors and review relevant literature.

A 21-year-old female with a history of vaping nicotine, cannabis use, and oral contraceptive pill (OCP) use presented with shortness of breath, left flank pain, and diarrhea. An initial evaluation suggested pyelonephritis, as supported by flank pain, pyuria, and bacteriuria on urinalysis, and a positive culture for *Escherichia coli*. Imaging of the abdomen and pelvis with non-contrast computed tomography (CT) revealed perinephric fluid with stranding of the left kidney and engorgement of the left renal vein. A follow-up contrast CT revealed a filling defect of the left renal vein. A CT angiogram was performed, showing a left renal vein thrombus.

Initial management consisted of broad-spectrum antibiotics (ceftriaxone) for suspected pyelonephritis and a multimodal pain regimen. Following the discovery of the renal vein thrombus, a multidisciplinary team, including vascular surgery, interventional radiology, hematology, and nephrology, initiated anticoagulation. They planned for thrombectomy if renal function deteriorated, as no acute kidney injury was evident through quantification of serum creatinine on initial presentation. Full-dose low molecular weight heparin was initiated and later switched to unfractionated heparin in preparation for thrombectomy. The patient experienced symptomatic improvement on anticoagulation, and renal function remained stable. A decision to cancel the thrombectomy and plan for outpatient oral anticoagulation with apixaban was made, given clinical improvement and stable serum creatinine concentration. Apixaban was continued for three months, and the patient experienced complete symptom resolution without recurrence at six-month follow-up.

This report focuses on an atypical case of RVT in the setting of a complicated UTI and multiple risk factors. Multiple explanations exist for thrombus formation, with a major driver being local endothelial damage secondary to infection. Another predisposing factor for RVT is nephrotic syndrome, which may have been secondary to infection or the renal thrombus itself. Additionally, OCPs and habitual nicotine use may have contributed to a hypercoagulable state and eventual thrombosis. Oral anticoagulation remains a safe, effective, and less invasive treatment of isolated renal vein thrombus without renal compromise.

## Introduction

Renal vein thrombosis (RVT) describes a thrombus formation in the renal vein or its branches [1]. The exact incidence of RVT is not known, but among those with nephrotic syndrome, estimates for occurrence range from 5% to over 60% [1]. The causes of RVT are complex, and its occurrence is rare without risk factors. A study of 218 patients with a first lifetime incidence of RVT evaluated at the Mayo Clinic over a 20-year period found that 143 patients, or 66.2%, had an underlying malignancy [2]. This was followed by 43 patients (19.7%) who presented with RVT in the context of nephrotic syndrome, most commonly membranous glomerulonephritis [2]. Less common etiologies include postoperative (6.5%), bacteremia (5.6%), urogenital infection (4.2%), pregnancy (2.7%), and oral contraceptive pills (OCPs) (0.4%) [2]. Other causes of RVT not directly measured in the study included kidney biopsy, extrarenal compression of the renal vein, decreased renal perfusion, renal transplantation, and inherited procoagulant defects [1,3].

The same study also found a bimodal age distribution at the first RVT presentation. The distribution of RVT spans the entire age spectrum from infancy to 85 years [2]. The first peak occurs in the initial decade of life, with a gradual increase in incidence until the second, more prominent peak in the seventh decade of life [2]. The average age at presentation is 55 years ± 19 years [2].

Clinical presentation of acute RVT can be nonspecific, with flank pain and gross hematuria being the most common symptoms [1-2]. In the study of 218 patients, 73% reported flank pain, followed by 36% of patients who experienced gross hematuria. Other symptoms less commonly reported were anorexia (21%), nausea/vomiting (13%), and fever (12%) [2]. Renal impairment was observed on laboratory findings in over half of patients; the average creatinine at the time of diagnosis was 1.5 ± 1.1 mg/dL [2].

The diagnosis of RVT is established through imaging. CT angiography is preferred for visualizing the renal veins if the patient’s estimated glomerular filtration rate is ≥30 mL/min/1.73 m² [1]. If CT angiography cannot be performed, such as in cases of renal impairment or contrast allergy, MR venography with gadolinium enhancement is a second-line imaging modality, offering similar sensitivity and specificity (92% and 100% for CT angiography compared with 94% and 100% for MR venography, respectively) [1]. Radiographic signs for RVT include renal vein enlargement, thickening of renal fascia, an edematous kidney capsule, and perinephric fat stranding [1].

The standard of care for management of RVT without acute kidney injury (AKI) is anticoagulation, typically with unfractionated or low molecular weight heparin [1]. Anticoagulation should be initiated early in the treatment course to prevent embolization [1]. In patients eligible for outpatient management with anticoagulation alone, a study of 21 patients with nephrotic syndrome found that switching to a direct oral anticoagulant (DOAC) such as rivaroxaban or apixaban resulted in no recurrence of thromboembolism [1].

In patients with AKI, thrombolytic therapy is favored, followed by maintenance on anticoagulation [1]. Local thrombolytic therapy has been reported to be effective with or without catheter thrombectomy [1].

RVT complicates at least seven cases of acute pyelonephritis reported in the literature [3]. Here, we present a case of RVT in a young patient with a complicated urinary tract infection (UTI) and pro-coagulation risk factors. We aim to examine the association between various risk factors and RVT formation in a young patient. We will highlight the importance of prompt recognition of RVT in patients with complicated UTI and multiple risk factors.

## Case presentation

A 21-year-old female presented to the emergency department with complaints of left-sided flank and generalized abdominal pain, shortness of breath, and diarrhea. Her past medical history was significant for longitudinal and current OCP use and anxiety. Her flank pain persisted for three days prior to presentation; her shortness of breath and gastrointestinal symptoms began the morning of presentation. She denied urinary frequency or dysuria, fever, chills, and weight changes. Her review of symptoms was otherwise negative. She denied a personal or family history of venous thromboembolism. Her social history was significant for a self-reported poor, sedentary lifestyle, excessive vaping, and cannabis use.

On arrival, the patient was alert and in moderate discomfort. She was afebrile with a temperature of 36.9°C, tachycardic at 112 beats per minute, normotensive at 121/82, and eupneic at 16 respirations per minute with an SPO2 of 98% on room air. Physical examination was notable for tenderness in the left lower quadrant of the abdomen and left flank with no costovertebral angle tenderness or peritoneal signs. An electrocardiogram showed sinus tachycardia. Laboratory findings revealed a normal white blood cell count and renal function. Urinalysis showed 3+ proteinuria, >180 RBCs, and pyuria with 74 WBCs. Urine culture revealed >100,000 colony-forming units of *Escherichia coli* (Tables 1-3).

**Table 1:** Laboratory results from blood chemistries ND: no data, HD: hospital day, eGFR: estimated glomerular filtration rate, AST: aspartate transaminase, ALT: alanine transaminase

Routine chemistry test	Result on HD#1	Result on HD#2	Result on HD#3	Reference range
Sodium	141	138	137	136-145 mmol/L
Potassium	4.6	4.2	4.4	3.6-5.2 mmol/L
Chloride	107	106	107	96-106 mmol/L
Bicarbonate	21	20	19	23-29 mEq/L
Glucose	91	112	94	72-99 mg/dL
Blood urea nitrogen	10	10	9	2.1-8.5 mmol/L
Creatinine	0.89	0.98	0.77	0.59-1.04 mol/L
eGFR	95	85	112	90-100 ml/min
Alkaline phosphatase	91	79	73	30-120 U/L
ALT	28	20	26	8-33 U/L
ALT	24	17	15	4-36 U/L
Total bilirubin	<0.2	<0.2	<0.2	0.20-2.1 mL/dL
Albumin	4.2	3.6	3.3	3.4-5.4 g/dL
Calcium	9.3	8.7	8.6	8.6-10.3 mmol/L
Anion gap	13	13	12	4-12 mEq/L
Lipase	45	ND	ND	0-160 U/L
Magnesium	ND	1.8	1.9	1.7-2.2 mg/dL
Phosphorus	ND	3.1	4.3	2.5-4.5 mg/dL
C-reactive protein	<0.30	ND	3.96	Below 3 mg/L

**Table 2:** Laboratory results from complete blood count ND: no data, HD: hospital day, WBC: white blood cells

Complete blood count test	Result on HD#1	Result on HD#2	Result on HD#3	Reference range
WBC	7.7	6.8	6.4	4.5-11 x 10^9/L
Hemoglobin	13.2	11.6	10.6	12-15.5 g/dL
Platelet count	314	207	211	150-400 x 10^3/µL
Hematocrit	40.9	35.5	32.6	36-44%
Erythrocyte sedimentation rate	18	ND	32	0-15 mm/Hr
C-reactive protein	<0.30	ND	3.96	<3.0mg/L

**Table 3:** Results of urinalysis and culture WBC: white blood cell, RBC: red blood cell, b-hCG: human chorionic gonadotropin, UA: urinalysis

Urinalysis	Hospital day 1
UA leukocyte esterase	Negative
UA nitrite	Negative
UA WBC	74/hpf
UA RBC	>180/hpf
UA bacteria	Trace
UA protein	3+
UA specific gravity	1.035
UA calcium oxalate crystal	4+
Culture	>100,000 CFU *Escherichia coli*
Qualitative b-hCG	Negative

**Figure 1:**
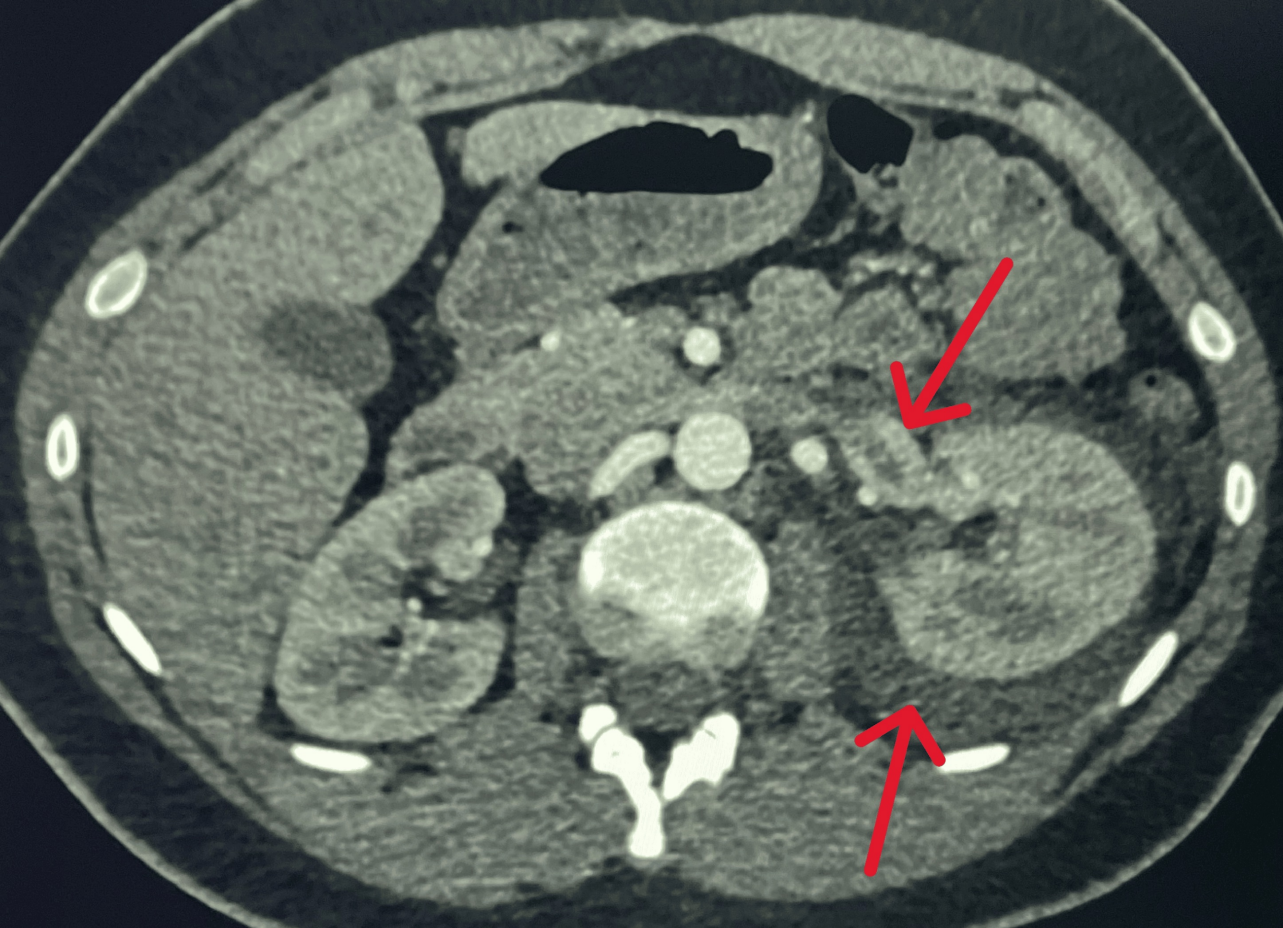
CT abdomen with contrast showing left renal vein engorgement and perinephric stranding The top red arrow indicates left renal vein engorgement, and the bottom red arrow indicates perinephric fat stranding. CT: computed tomography

A non-contrast CT of the abdomen and pelvis identified perinephric fat stranding and engorgement of the left renal vein (Figure 1). The patient was given a 1-liter bolus of 0.9% normal saline and continued on maintenance fluids at 75 milliliters an hour. A follow-up contrast-enhanced CT of the abdomen and pelvis confirmed a left renal vein filling defect and RVT.

The patient was admitted and initiated on enoxaparin (1 mg/kg) for her RVT and empiric IV ceftriaxone for her UTI. A multimodal pain regimen was given as needed for pain. Her tachycardia had resolved following her bolus of normal saline and initiation of pharmacologic pain management. Oral combined contraceptives were discontinued.

On hospital day two, anticoagulation was transitioned from enoxaparin to continuous heparin infusion and titrated to goal activated partial thromboplastin time per hospital protocol. Vital signs remained stable (BP 103/70; HR 87; RR 16; T 36.6ºC; SpO2 97%), and renal function remained within normal limits. The patient experienced symptomatic improvement of her shortness of breath and abdominal pain. Daily clinical evaluations and repeat metabolic and cell counts were reassuring, showing no evidence of disease progression.

On hospital day three, no changes were made to her treatment regimen, and her renal function remained stable. She remained hemodynamically stable and asymptomatic. Hematology was consulted, and the patient was scheduled to start apixaban the following day for outpatient anticoagulation.

On hospital day four, the patient was transitioned to oral anticoagulation with apixaban and received her first dose of 5 milligrams. The patient was discharged later that day with instructions to continue apixaban 5 milligrams twice daily until further notice. She was prescribed a seven-day course of cefdinir 300 milligrams twice daily for her UTI. Her combined OCP was switched to a progestin-only contraceptive. She was scheduled to follow up with hematology to review her hypercoagulable workup and assess the duration of anticoagulation. A follow-up was planned with vascular surgery to undergo a repeat CT abdomen and pelvis in two weeks to ensure resolution of her RVT.

Follow-up

The patient received anticoagulation with apixaban for a total of three months. The patient deferred her two-week follow-up CT imaging due to concerns about radiation exposure. A renal ultrasound was pursued at a one-month follow-up, revealing normal anatomy without dilatation of the left renal vein. She was seen by hematology two weeks after discharge; her hypercoagulable workup was unremarkable. At her three-month follow-up, apixaban was discontinued. A urinalysis at a four-month follow-up showed normal findings without proteinuria. Since her hospitalization, she has made positive lifestyle changes, including smoking cessation, adhering to an exercise regimen, and healthy eating practices. At six months, she reported complete resolution of her condition without recurrence.

## Discussion

RVT is an uncommon but clinically important entity that is often underdiagnosed due to its nonspecific presentation and overlap with more common urologic or abdominal pathologies [1]. Venous thrombus formation can generally be attributed to the pillars of Virchow’s triad: blood flow stasis, endothelial injury, and hypercoagulability [1,4-6]. Hypercoagulable states associated with nephrotic syndrome, malignancy, and trauma increase the risk of RVT [1,4,6-8]. In this case, an RVT occurred in a 21-year-old female with no overt thrombophilia and multiple potential contributory risk factors.

The association between RVT and states of renal inflammation has been documented in the literature. Infection-associated inflammation, particularly complicated UTI or pyelonephritis, can cause local endothelial injury and systemic inflammatory cytokine release, further contributing to hypercoagulability [1,4-6]. Inflammatory states promote thrombosis through increased platelet activation and disruption of the vascular endothelium [1,4-6]. Other risk factors for the patient’s thrombophilia include OCP use, smoking, and nephrotic-range proteinuria [5,9-10].

Nephrotic-range proteinuria is one of the most well-established predisposing conditions for RVT [1,5,8]. Nephrosis leads to urinary loss of anticoagulant proteins such as antithrombin III and increased hepatic production of procoagulant factors, tipping the balance toward a thrombotic state [1,4-5,8]. A formal diagnosis of nephrotic syndrome was not made in this case; however, proteinuria >3.5 g/day is the diagnostic threshold for nephrosis. The presence of 3+ proteinuria on urine dipstick identifies nephrosis as a potential risk factor for RVT in this case, although proteinuria was not quantified [1]. A serum albumin <2.9 g/dL has been associated with increased hypercoagulability and thrombotic risk [1,7-8]. However, this patient had proteinuria without hypoalbuminemia until later in her hospitalization, and serum albumin at its lowest was 3.3 mg/dL. Therefore, we attribute the patient's hypoalbuminemia to hemodilution secondary to the administration of IV fluids.

OCP use is a well-documented, dose-dependent risk factor for VTE, particularly in young women [1,9,11]. Estrogen enhances coagulation by increasing levels of fibrinogen and factors VII, VIII, and X, while reducing levels of protein S, a natural anticoagulant. Combined with other risk factors, its thrombogenic potential is amplified [9,11]. Progestin-only contraceptives confer a reduced risk of VTE, particularly in the setting of prothrombotic risk factors [9-10].

Nicotine use, whether through smoking or vaping, increases thrombotic risk by inducing platelet activation, increasing serum fibrinogen, and promoting endothelial dysfunction [10-12]. While often underemphasized in young populations, nicotine’s role in promoting both arterial and venous thrombosis has been increasingly recognized in literature, with risk being positively correlated with chronicity of use [10-12]. Epidemiologic data demonstrate that current smoking increases the risk of venous thrombosis (odds ratio (OR) 1.43, 95% confidence interval (CI95) 1.28-1.60), with the effect most pronounced in young women who also use OCPs (OR 8.79, CI95 5.73-13.49) [10].

Standard thrombophilia testing in this case was unrevealing; however, this does not exclude the presence of rarer or undetected prothrombotic conditions [13-15]. Further follow-up with hematology is essential to evaluate underlying glomerular disease and assess the need for extended anticoagulation or treatment of underlying conditions [12-15].

This case illustrates the clinical complexity of RVT and reaffirms the importance of advanced imaging in ambiguous flank pain, particularly when the initial workup is inconclusive [1-3,6]. Our patient presented with generalized abdominal pain, left flank pain, shortness of breath, and diarrhea: nonspecific symptoms that initially mimic more common diagnoses such as pyelonephritis or nephrolithiasis [1-3,6]. Urine studies were notable for hematuria, pyuria, proteinuria, and a positive culture for *Escherichia coli*, suggestive of infection [1,4-5]. Despite her urinalysis, the patient presented afebrile and without urinary symptoms. She was found to have a white blood cell count within normal limits, making pyelonephritis a less convincing diagnosis and indicating CT imaging to discern her condition from other intra-abdominal pathologies [13,16-17].

CT angiography provides a timely and definitive diagnosis for suspected intra-abdominal venous thromboembolism [1,12-13,16]. While Doppler ultrasound offers a non-invasive alternative, its sensitivity for detecting RVT, especially segmental or early thromboses, can be limited [1,12,16]. CT and MRI remain superior for evaluating renal vasculature and guiding treatment decisions [1,13,16].

Anticoagulation was initiated with low molecular weight heparin, followed by continuous heparin infusion as a precaution to renal dysfunction associated with RVT [12,14,16,17-19]. The patient was transitioned to a DOAC, apixaban, due to decreased need for routine monitoring and lower risk of major bleeding as seen in meta-analyses and randomized clinical trials [14,17-19]. Furthermore, studies have supported the efficacy and safety of DOACs in unusual-site venous thromboembolism [17,20]. The patient’s stable renal function and absence of inferior vena cava involvement favored conservative management [17,20].

## Conclusions

This case highlights the multifactorial nature of RVT development in a young adult female. It demonstrates that even in the absence of classical hereditary thrombophilia or malignancy, a confluence of transient and chronic risk factors, including proteinuria, UTI, hormonal therapy, and nicotine exposure, can culminate in significant thrombotic events. Clinicians must maintain a high index of suspicion for RVT in young patients with flank pain and ambiguous findings, particularly when multiple subclinical risk factors coexist. This case supports a more nuanced understanding of thrombotic risk in younger populations. Furthermore, it reinforces the need for a multidisciplinary approach to diagnosis, treatment, and long-term monitoring of atypical site venous thromboembolism in unsuspecting populations.
